# An Overview of the Elusive Passenger in the Gastrointestinal Tract of Cattle: The Shiga Toxin Producing *Escherichia coli*

**DOI:** 10.3390/microorganisms8060877

**Published:** 2020-06-10

**Authors:** Panagiotis Sapountzis, Audrey Segura, Mickaël Desvaux, Evelyne Forano

**Affiliations:** 1 Université Clermont Auvergne, INRAE, UMR 0454 MEDIS, 63000 Clermont-Ferrand, France; dkause@chr-hansen.com (A.S.); mickael.desvaux@inrae.fr (M.D.); evelyne.forano@inrae.fr (E.F.); 2 Chr. Hansen Animal Health & Nutrition, 2970 Hørsholm, Denmark

**Keywords:** cattle, STEC colonization, microbiota, bacterial interactions

## Abstract

For approximately 10,000 years, cattle have been our major source of meat and dairy. However, cattle are also a major reservoir for dangerous foodborne pathogens that belong to the Shiga toxin-producing *Escherichia coli* (STEC) group. Even though STEC infections in humans are rare, they are often lethal, as treatment options are limited. In cattle, STEC infections are typically asymptomatic and STEC is able to survive and persist in the cattle GIT by escaping the immune defenses of the host. Interactions with members of the native gut microbiota can favor or inhibit its persistence in cattle, but research in this direction is still in its infancy. Diet, temperature and season but also industrialized animal husbandry practices have a profound effect on STEC prevalence and the native gut microbiota composition. Thus, exploring the native cattle gut microbiota in depth, its interactions with STEC and the factors that affect them could offer viable solutions against STEC carriage in cattle.

## 1. Introduction

The domestication of cattle, approximately 10,000 years ago [1], brought a stable supply of protein to the human diet, which was instrumental for the building of our societies. As the earth’s population continued to grow, nutritional demands increased, pushing us towards industrial farming approaches, which can increase meat and dairy production. These approaches include the use of high energy feeds (e.g., starchy grain) [2]; the use of distillery waste products as feed [3]; the use of growth-promoting antimicrobials in several non-EU countries [4]; and the use of crowded industrialized farms [5]. The use of feedlots, which is a common practice in the US and Canada, even though it increases the density of animals per square meter, favors the spread of zoonotic foodborne pathogens [5]. High energy feeds, administered to the animals in order to increase weight gain, also increase the risk of meat and dairy contamination with foodborne bacterial pathogens [6].

One of the most dangerous foodborne pathogens that is occasionally present in cattle GIT is the enterohemorrhagic *Escherichia coli* serotype O157:H7. The serotype O157:H7 belongs to the enterohemorrhagic *Escherichia coli* (EHEC) group and is part of the bigger group of Shiga toxin-producing *Escherichia coli* (STEC). EHEC serotypes are typically defined by their ability to cause disease in humans, hence all STEC are potential pathogens, but unless they have demonstrated pathogenicity to human hosts, they are not classified as EHEC [7,8,9,10]. EHEC serotypes O157:H7, O26:H11, O145:H28, O103:H2, O111:H8 and others pose a significant threat to human health [9,11] and their presence in food products has resulted in epidemics in Europe, the US and Japan [12,13,14]. The serotype O157:H7 alone is estimated to be responsible for approximately 405 million USD loss every year [15]. Cattle are the primary reservoir of STEC and healthy asymptomatic carriers that can occasionally excrete STEC in their feces. Animals that excrete >10^4^ STEC CFU/g feces are known as “super-shedders” [16,17]. Strategies to limit STEC in dairy farms include vaccination, bacteriophages, feed additives and direct-fed microbials (DFMs) [18,19]. DFMs offer a promising alternative, as they have not only been implicated as important agents preventing the colonization of *E. coli* O157:H7 [18,20,21]; they also play important roles in host nutrition, as they can protect against ruminal acidosis and they have beneficial immunomodulatory effects [22,23]. However, only a few DFMs have demonstrated efficiency in reducing the shedding of STEC O157 under commercial farm conditions [21] and new candidates are needed.

This review aims to gather existing knowledge in the areas of STEC carriage in bovine hosts and the strategies to control it, but also to highlight the potential of the native microbial symbionts of cattle to prevent zoonotic STEC from colonizing the gastrointestinal tract (GIT). Thus, we present literature on STEC carriage in bovine hosts, but also relevant information on members of the native GIT bacterial community, bacteriophages and ciliate protozoa, which are the groups that offer the greatest potential for inhibiting STEC. We discuss the factors that affect the composition of microbial symbionts and STEC prevalence and present the methods that are currently used to prevent STEC carriage. By comparing STEC and GIT microbiota and discussing the underlying mechanisms that affect their prevalence and abundance, we attempt to highlight the entangled, often hidden relationships between STEC and microbiota, which we could potentially use to prevent STEC carriage.

## 2. STEC: The Elusive GIT Passenger

### 2.1. STEC Definition and Pathology

The common pathogenic *E. coli* strains that can cause enteric disease in humans, are classified in seven pathotypes: the enteropathogenic *E. coli* (EPEC), the Shiga toxin-producing *E. coli* (STEC), the enterotoxigenic *E. coli* (ETEC), the enteroinvasive *E. coli* (EIEC), the enteroaggregative *E. coli* (EAEC), the diffusely adherent *E. coli* (DAEC) and the adherent invasive *E. coli* (AIEC), although the pathogenicity of AIEC remains controversial [9,24,25,26]. EHEC causes symptoms that range from mild gastroenteritis to hemorrhagic colitis and hemolytic uremic syndrome (HUS). From a genotypic point of view, EHEC belongs to the larger group of STEC as they all harbor the Shiga toxin gene (*stx*). More than 400 serotypes have been characterized to date and more than half of them have been associated with serious enteric disease [13]. While the virulence level of STEC is considered to be variable, ranging from avirulent to hypervirulent strains [7,8], reports suggest that all STEC have the potential to be pathogenic to humans [9,25,27]. The *E. coli* O157:H7, which is possibly the most well studied serotype, is widely accepted as the etiological agent of many outbreaks: 64% of the HUS cases in Europe between 2002 and 2006 and 60.4% of the cases between 2012 and 2017 were attributed to O157:H7. Other non-O157 strains (e.g., *E. coli* O26:H11, O145:H28, O103:H2, O111:H8) have also been recognized as equally important outbreak agents [9,25].

In the US, O157 and non-O157 STEC strains are estimated to be responsible for more than 175,000 cases annually [25] and are the most common cause of acute renal failure in children [28]. In 2017 in France, 164 cases of children (<15yo) with HUS were detected and the number of cases has almost doubled in the last 20 years [29]. Infections can be caused by only a few cells [30] and even though they can be asymptomatic, in many cases, they are characterized by watery and/or bloody diarrhea, HUS, acute renal failure, microangiopathic hemolytic anemia, and thrombocytopenia [31,32]. The kidneys, the central nervous system, the lungs, the pancreas, and the heart can be affected, with children and the elderly being the most susceptible groups [31]. Various methods have been developed to characterize STEC infections with PCR of STEC virulence genes being possibly the most popular [33]. Even though the number of annual cases is not very high, STEC infections are considered an important public health issue because treatment options are limited: administration of antibiotics is not recommended because it can lead to toxin production and promote the development of HUS [18].

### 2.2. Virulence Factors

**Figure 1:**
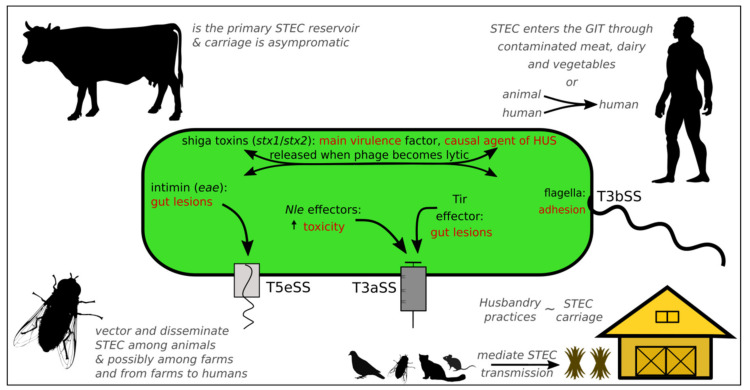
An overview of STEC virulence factors, transmission agents and threat to humans. A STEC cell is illustrated at the center of the figure. Inside the cell, the main virulence factors (black font) and the phenotypes they contribute to (red font) are presented. The primary STEC reservoir is cattle (top left) and contamination occurs through consumption of contaminated meat, vegetables and dairy products and contact with animals carrying STEC or infected humans. *Musca domestica* (the common housefly; bottom left) is a key insect vector for STEC transmission in farms, to vegetables and potentially to humans. Other known STEC vectors include birds, rodents, cats, dogs and ruminants (bottom), but very little research has been conducted in this area [25,39]. STEC levels in cattle vary depending on the season, country and animal husbandry practices (bottom right). Silhouette images were modified from phylopic (phylopic.org), available under a Public Domain License.

The repertoire of genes encoding virulence factors in STEC strains is largely similar, however, the gene organization can be vastly different on the grounds that they are located in prophages, plasmids, pathogenicity islands (PAIs) and other mobile elements [25]. The locus of enterocyte effacement (LEE) is a PAI that contains many key pathogenic genes such as the necessary components of the Type III secretion system (T3SS), the effector molecules, the translocated intimin receptor (Tir), and the intimin (*eae*). Together, they are responsible for the attaching and effacing (A/E) lesions induced in intestinal epithelial cells [9,25,27,34,35] (Figure 1). 

The defining virulence genes (also used as biomarkers) are the *stx* genes: two *stx* genes have been identified, *stx1* and *stx2,* producing four (Stx1a, Stx1c, Stx1d and Stx1e) and twelve subtypes (Stx2a–Stx2l), respectively [9,36]. Out of the two main variants, Stx2 is more diverse and associated with higher pathogenicity [9,25,37]. Stx is only expressed when the phage becomes lytic, during which the STEC host is lysed and the Stx toxins are released [37] (Figure 1). The released toxins can subsequently bind to the globotriaosylceramide receptor (Gb3), which is present in some mammalian cells, and induce cell death. Other virulence agents include the Nle effectors [38], which aid in pathogenicity by promoting colonization, inhibiting the host immune system and prolonging infection. Additionally, considering that the most virulent strains have the complete set of *nle* genes, they are likely key virulence factors [25] (Figure 1). 

### 2.3. Cattle Are the Main Reservoir of STEC (but in Low Abundance)

Cattle are one of the sources of human STEC infections and many outbreaks have been linked to consumption of undercooked beef or unpasteurized dairy products [12] (Figure 1). Other sources of infection include contaminated vegetables and contact with animals carrying STEC or infected humans [9] (Figure 1). STEC can be present in sheep, wild deer, goats, pigs, pigeons, cats, dogs, rats and rabbits [25], however, cattle are the primary reservoir of STEC O157 and non-O157 serogroups [17,31]. 

Cattle, like other ruminants (e.g., sheep, goats), are asymptomatic carriers as they lack the vascular receptors (Gb3) for Stx toxins [33]. Therefore, it has been suggested that the aforementioned virulence factors (Stx, Tir, Eae, Nle) may be relevant for a symbiotic relationship with the adult host [40,41,42,43]. Stx can affect functions of T- and B-lymphocytes, suggesting that they could modulate the immune response with possible consequences on STEC survival and colonization of the bovine GIT [44]. STEC immunotolerance in cattle has been attributed to a potential interplay between Shiga toxin and host cells and/or the inability of STEC antigens to reach the immune response sites [45]. It has also been hypothesized that Stx could increase the rate of survival of *E. coli* O157:H7 in the presence of grazing protozoa in the bovine GIT, although such evidence remains contradictory and deserves further investigation [46,47]. 

STEC abundance is low in cattle; the total amount of *E. coli* cells (which includes STEC) typically accounts for less than 0.01% of the total microbial population in the rumen [48], while in the cecum and feces, *E. coli* accounts for 0.1–1% of the total microbial population [49]. Within farms, the principal mode of *E. coli* O157:H7 transmission among animals is through contact with each other, contaminated feces, feed or water and through houseflies, which are an important *E. coli* O157:H7 vector [19] (Figure 1). STEC can survive in manure and pen floors for periods of up to four months or more [18,50,51,52], which suggests a high prevalence in the farm environment. Only a few STEC cells are sufficient to colonize a new host [53] and STEC can grow well in open ecosystems [30] and successfully transmit from open environments to new hosts [54]. On the other hand, vertical transmission has not been demonstrated so far, as in an earlier study it was demonstrated that STEC shedding cows had different STEC strains than their calves [55]. Thus, more studies are needed to examine whether STEC can also transmit from mother to offspring, or rather strictly from the environment. 

Estimating accurately the prevalence of STEC in cattle is challenging for three reasons: (i) there is no universal method to identify all STEC serotypes in cattle [25,33], (ii) studies have been performed in different conditions, seasons, locations and animals, and (iii) there is a large variability in prevalence. The prevalence can vary from one study to another within a country, and also between countries. A meta-analysis estimated the prevalence of *E. coli* O157 in cattle at the world global level at 5.68%, a higher prevalence being found in US, Australia, Japan and Italy, and the lowest (<1.25%) in Brazil, France, Norway and Germany [56]. Younger animals are more likely to carry *E. coli* O157:H7 [57,58,59] and non-O157 STEC serotypes [57,60]. This could be related to a less developed immune system, ruminal function and GIT microbiota, as suggested by Zhao [61]. Other potential contributing factors include the preweaning diet and parturition stress, which can lead to increased STEC shedding [60]. Seasonal variation plays an important role as O157:H7 prevalence typically peaks during the summer and the beginning of autumn [12,58,62], while winter conditions seem to favor some non-O157 (i.e., O111 and O145) strains [62]. Seasonal variation is likely related to the temperature and the host physiological response to changing seasons, which further affects STEC transmission, as seasonal changes can trigger enhanced STEC shedding which facilitates rapid spread to new hosts [48]. However, since seasonal variability affects members of the native microbiota as well [63], STEC seasonal variation may also be related to increased/reduced microbial competition. Diets, fasting and farm practices also have a significant effect on the prevalence of STEC in cattle (see below).

### 2.4. STEC Survival, Colonization and Metabolism in the Bovine GIT

**Figure 2:**
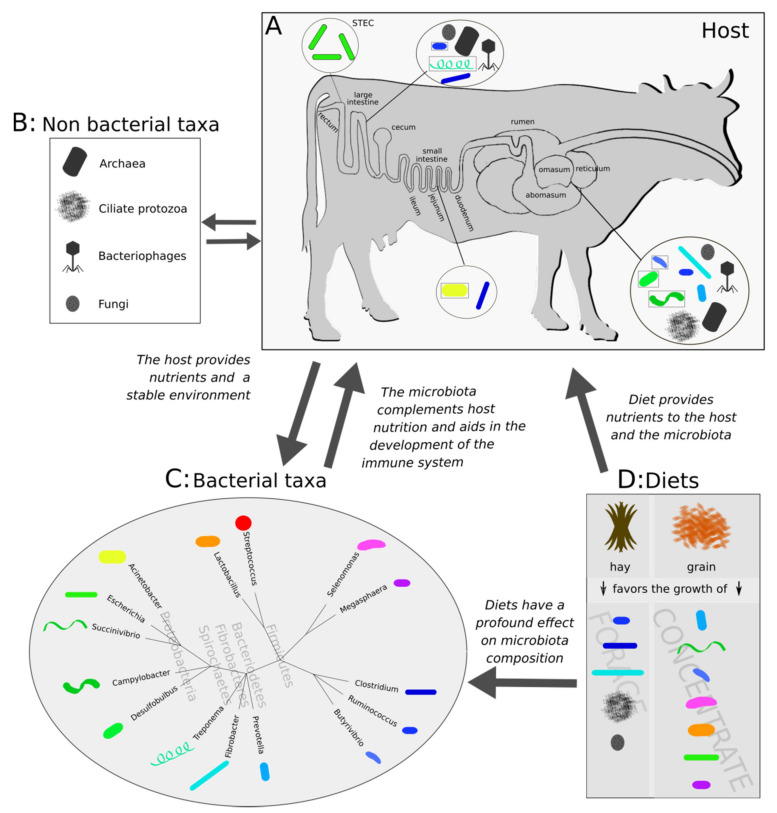
Summary of the most common bacterial and other taxa found in the cattle GIT, their tissue localization and the effect of host diet on their abundance. **A**: A graphic illustration of the cattle gastrointestinal tract compartments. The black circles illustrate the associated microbial communities. Within each circle, the most common genera are presented. Colored illustrations represent bacterial taxa, black and gray illustrations represent microbial taxa and bacteriophages. Black squares around bacterial illustrations indicate that they are part of the mucosa, while bacterial cells with a black outline (top left) indicate the STEC group, which colonizes the RAJ. **B**: Summary of nonbacterial taxa in the GIT. **C**: Bacterial taxa and their taxonomic relationships presented in a phylogenetic tree. The tree is built using the common tree function (www.ncbi.nlm.nih.gov/Taxonomy/CommonTree/wwwcmt.cgi). Phylum names are shown in light gray font behind tree branches. **D**: Association between diet and microbial abundance. Silhouette images were modified from phylopic (phylopic.org), available under a Public Domain License or drawn in Inkscape 0.92.5.

Once ingested by the bovine host, STEC is able to survive, persist and colonize the bovine GIT. *E. coli* O157:H7 transits through the rumen, which is neither a colonization nor a proliferation site as opposed to the intestine (ileum, jejunum, caecum, colon and rectum) [64,65,66]. Even though not all STEC cells are able to resist the stress induced (acid, low oxygen, osmolytes, low nutrients availability) by the harsh environment of the rumen, some cells are able to persist through a well-regulated response [67,68]. In order to resist the low pH of the abomasum, *E. coli* O157:H7 uses a specialized glutamate dependent acid resistance system, encoded by *gadABC.* Additional resistance systems, such as the Arg system, likely contribute [68], as the complexity of O157:H7 adaptation to low pH is not yet entirely understood [69,70]. Following passage through the rumen, *E. coli* O157:H7 is able to resist the bile [71,72] in the small intestine [73], even though not all STEC are able to do so [71], and reach the rectoanal junction (RAJ) mucosa, which is the principal colonization site [66] (Figure 2).

STEC can express numerous molecular determinants involved in surface colonization along the bovine GIT, such as cell surface proteins [74,75,76]. The T5eSS, Intimin and Tir effector together with the T3aSS EscF-EspABD (which is the molecular structure encoded by the LEE4 operon forming the injectisome) are involved in the formation of A/E lesions localized at *E. coli* O157:H7 microcolonies in RAJ mucosa [77,78] (Figure 1). The injectisome, Tir and Intimin are also required for the colonization of the large and small intestine of cattle as A/E lesions have been observed in the mucosa of the ileum and colon [79,80]. These molecular determinants are globally important surface colonization factors for STEC carriage in ruminants [81,82,83]. Flagella likely initiate bacterial adhesion to the intestinal epithelium; H7 (flagella) promotes adhesion to rectal epithelium, contrary to H6, H11 and H21 [84], even though both H7 and H6 can bind to bovine mucus (mucins I and II) [85]. Flagella expression (T3bSS) diminishes after the initial attachment and LEE-encoded components take over [84]. While F9 (fimbriae 9) is clearly involved in the adhesion to rectal epithelial cells [86], the contribution of other pili is likely [87], but hard experimental evidence for the type 1 pili, type 4 HCP (hemorrhagic coli pili), LPF (long polar fimbriae), curli, ECP (*E. coli* common pili), ELF (*E. coli* laminin-binding fimbriae), or SFP (sorbitol fermenting fimbriae) is still lacking [74,88]. Besides multimeric adhesins (injectisome, flagella and pili), the monomeric adhesin EspP (extracellular serine protease plasmid-encoded), which belongs to the T5aSS and is encoded by plasmid pO157, is involved in the adhesion to rectal epithelial cells [77]. Knowledge of the exact role of Intimin in the colonization of the bovine intestine is still lacking, but it could have a similar role to the one that has been described in humans; Intimin exists in 38 distinct subtypes [89] that influence the site of intestinal colonization in humans in a Tir-independent manner, and Intimin appears to restrict colonization in human follicle-associated epithelium [90]. STEC carries additional monomeric adhesins but in-depth investigations are needed to determine their potential involvement in the colonization of the bovine GIT. Some of these adhesins are: the Eha (enterohemorrhagic *E. coli* autotransporters), the Saa (STEC autoagglutination adhesin), the Ag43 (antigen 43), the Sab (STEC autotransporter mediating biofilm formation), the Efa-1 (*E. coli* factor adherence 1), the OmpA (outer membrane protein A) and the Iha (iron-regulated protein A homolog adhesin) [74,76].

## 3. The Native GIT Microbiota and Its Interactions with STEC

### 3.1. The Diverse GIT Microbial Community

In order to reach the RAJ, STEC must first transit through the bovine GIT, which is colonized by native microbial symbionts. The rumen is the first GIT compartment STEC enters and is mainly colonized by Bacteroidetes (i.e., Prevotella) and Firmicutes (*Ruminococcaceae*), whose main role is to catabolize complex plant polysaccharides [91,92,93,94,95]. Lactic acid bacteria (LAB; i.e., lactobacilli, streptococci) constitute a smaller part of the rumen community in forage-fed animals [96], but are prominent members in early life and in grain-fed animals [96,97], and they have a demonstrated inhibitory effect against STEC (see below). Ciliate protozoa such as *Epidinium*, *Polyplastron* and *Entodinium* digest structural and storage carbohydrates [98], promote homeostasis by stabilizing the pH in the rumen [99,100], and they can interact with STEC in cooperative and competitive ways (see below). Fungi and archaea are also part of the rumen ecosystem but no studies to our knowledge have suggested an interaction with STEC. Fungi are efficient fiber decomposers and are likely related to xylan and cellulose degradation [98,101,102] and initiation of feed breakdown [101]. Archaea use the hydrogen produced during fermentation to produce methane and prevent H_2_ accumulation in the rumen that would inhibit fiber digestion [98,103]. Bacteriophages in the rumen typically belong to the order of Caudovirales [104] and they have been associated with the dominant Firmicutes and Bacteroidetes but also Proteobacteria including STEC [98,101]. The three main roles proposed for the rumen bacteriophages are: (i) the nutrient and enzyme turnover as a result of bacterial cell lysis, (ii) the regulation of dominant species of the bacterial population, and (iii) the horizontal transfer of genes among bacterial species [104].

In the intestine, members of the *Clostridiaceae* family are common in the digesta, while *Acinetobacter*, *Treponema* and *Ruminococcaceae* are rather found in the mucosa, which is also the principal colonization site of STEC [66,105,106,107,108,109] (Figure 2). The epimural microbial communities have been suggested to play a key role in the development and homeostasis of the GIT immune system (especially in early life) [22,98,110]. In addition, they may potentially interact with STEC, since several of these taxa have been identified as over/underrepresented in cattle carrying STEC (Table 1). LAB are also prominent members of the intestinal microbiota as they are dominant in early life calves [111] and appear to protect against gut dysbiosis [110,112]. Both LAB and bacteriophages (of the Caudovirales order) are often isolated from cattle feces and are known to inhibit STEC [113,114] (see below). Other members of the intestinal microbiota include anaerobic fungi and the methanogenic genera *Methanobrevibacter* and *Methanosphaera,* commonly identified in cattle feces [98,105,115], but while their role in the rumen and intestine is often assumed to be similar, there is no experimental evidence whatsoever of their function in the intestine. 

### 3.2. Interactions between STEC and Native Bacterial Symbionts

In order for *E. coli* O157:H7 to survive and persist in the GIT, it is necessary to either avoid conflict, outcompete or exploit the native microbiota. STEC is able to survive and persist in the GIT through a well-regulated acid resistance system (gadABC). The aforementioned GAD resistance system is activated by the rumen microbiota AHLs signals, which trigger the downregulation of the LEE system and the upregulation of the GAD system. This allows *E. coli* O157:H7 to withstand the low pH in the abomasum [121,122]. *E. coli* O157:H7 relies on the metabolism of galactose, N-acetylglucosamine (GlcNAc), N-acetylgalactosamine (GalNAc), fucose, mannose, and N-acetylneuraminic acid (Neu5Ac). These nutrients are derived from the turnover of enterocytes and the degradation of mucins by mucinolytic symbionts and constitute the carbohydrate portion of the mucus covering the epithelium of the intestine [123]. *E. coli* O157:H7 is able to catabolize in vitro many of these compounds faster than the native microbiota (mannose, GlcNAc, Neu5Ac and galactose preferentially) [123]. Ethanolamine, a carbon and nitrogen source that is abundant in the GIT, as a result of the constant turnover of eukaryotic and bacterial cells, seems to be of particular importance for the colonization of *E. coli* O157:H7. It likely provides a nutritional advantage to STEC because it may not be the preferred substrate for the GIT microbiota [124]. Similar to ethanolamine, aspartate is a key compound for *E. coli* O157:H7 colonization, as mutant assays have demonstrated that it gives *E. coli* O157:H7 a competitive advantage [125]. Similarly, it has been suggested that serine, glycerol and lactate may also be preferred substrates for STEC [126]. The aforementioned nutrients are all present in the GIT [127,128] and although they are not the most abundant nutrients (e.g., GalNAc>fucose: [128]), they are selectively catabolized by *E. coli* O157:H7 [123,125,126,129]. This selective catabolism is possibly part of a niche nutrient specialization that allows *E. coli* O157:H7 to reduce or avoid microbial competition (by catabolizing the less ‘popular’ nutrients) with native GIT endosymbionts. The native GIT microbiota is adapted to the breakdown of complex polysaccharides [94,102], unlike *E. coli* O157:H7, which has a preference for mucus-derived sugars [123,130,131] (a strategy that has also been reported for *Vibrio cholerae* and *Campylobacter jejuni* [123]) and scavenging of other carbon and nitrogen sources (e.g., amino acids, endogenous glycerol [131]). The choice of the nutrients is usually dependent on the growth phase [129,131] and the GIT compartment. Genes encoding proteins involved in the metabolism of ethanolamine are upregulated in STEC O157:H7 in rumen and the small intestine, while those involved in the metabolism of mucus-derived carbohydrates are upregulated in the small intestine and rectum [131]. Nevertheless, the ability of *E. coli* O157:H7 to metabolize these compounds does not always guarantee its survival, as other microbes may be present that can metabolize the same compounds faster or produce antibacterial molecules. For example, *E. coli* O157:H7 can catabolize glycerol [126,131]. *Limosilactobacillus reuteri* (previously known as *Lactobacillus reuteri;* [132]) can also catabolize it and in the process of doing so, it produces HPA that inhibits *E. coli* O157:H7 [20]. 

### 3.3. STEC Predation by Phages and Protozoa

Phages targeting *E. coli* O157:H7 have been isolated from humans, animals and other environments where STEC is present [133,134,135,136], suggesting a predator–prey relationship. Phages of the *Myoviridae* and *Siphoviridae* families (Caudovirales order) that specifically target *E. coli* O157:H7 have often been isolated from cattle feces [114]. T4-like *Myoviridae* phages have been found in higher titers in low shedding animals, suggesting that their presence is linked to reduced O157:H7 titers [114]. Recent studies have further suggested that cattle intestinal endemic phages target specific STEC strains: *Myoviridae* and *Siphoviridae* phages target various non-O157 groups [137]; *Myoviridae* phages (isolated from cattle feces) target O177, O157, O26 STEC serogroups [138]; a T4-like phage of the *Myoviridae* family isolated from the cattle intestine (not from feces) targeted O157:H7 and non-O157 serotypes but not *Salmonella* strains [139]. Ciliate protozoa have been reported to predate on STEC, but predation does not appear to be STEC specific [47]. In sheep, different species of ciliate protozoa have different interactions, as *Epidinium spp.* appear to have a predatory relationship with STEC, while *Dasytricha spp*. may serve as hosts to STEC endosymbionts [140]. On the other hand, co-cultures of ciliate protozoa of the genera *Entodinium* and *Epidinium* and STEC showed no interaction between them [141].

## 4. STEC Emergence in Cattle Farms

### 4.1. STEC and GIT Symbionts Are Affected by Diet

Dietary change has a profound effect on STEC shedding: distillers grain (DG) has demonstrated a clear effect in increasing O157:H7 shedding [48,142], even though non-O157 STEC serotypes may respond differently [143] and isolated studies have challenged this finding [144]. Barley-feeding and steam-flaked grain (as opposed to dry-rolled grain) have also been associated with increased O157:H7 incidence [48]. 

Diet also has a profound effect on the GIT microbial communities: unclassified Bacteroidales, unclassified Clostridiales, *Ruminococcaceae* and *Fibrobacter* are more abundant in forage-fed bovines; *Prevotella* and unclassified *Succinivibrionaceaea* are enriched in animals fed diets containing concentrate and *Butyrivibrio* in animals fed mixes of forage and concentrate [115] (Figure 2). *Ruminococcus*, one of the dominant bacteria, is evenly distributed between animals fed forage and concentrate, but this is an exception [115]. When animals are fed high grain diets, lactic acid producing (e.g., *S. bovis*, lactobacilli) and lactic acid utilizing taxa (*Megasphaera elsdenii*, *Selenomonas*) increase [22,96]. In contrast, rumen protozoa and anaerobic fungi tend to have lower concentrations in high grain-fed animals, but not always (e.g., cows with diet-induced ruminal acidosis can have a higher abundance of rumen fungi than healthy cows) [98], while the starch-degrading *Entodinium* becomes dominant in high grain diets [101]. Microbiota composition is also affected by feed supplements [145,146], periodical starvation [147] and high fat diets [148].

### 4.2. Ruminal Acidosis and the Emergence of Acid Resistant Strains

Transition from high-forage to high-concentrate feed can trigger a condition called (sub)clinical acidosis, which is characterized by low ruminal pH (<5.2 or <5.6; the definition varies among studies), increased lipopolysaccharide presence, inflammation and other host-specific negative effects [149]. The condition is commonly distinguished as either a severe (clinical acidosis) or a less severe response (subclinical acidosis), also known as acute or subacute ruminal acidosis (SARA). SARA is often characterized by reversible rumen pH depressions and the production of other toxic biomarkers that can precipitate further health disorders [99]. The effect on microbial communities is more severe in the lumen than the epithelium of the rumen [150,151] and microbial communities in the intestine are affected as well [99]. In the rumen, SARA results in a reduction in microbial diversity and richness that may signify a reduction or shift in functionality and catabolic potential [99]. At the phylum level, a general decrease in Bacteroidetes and Fibrobacteres [150,152,153] and an increase in Firmicutes [99] is observed. An increase in *Prevotella* (both in rumen and rectum) and *S. bovis* (in rumen) has been documented in many studies [150,152,153,154,155,156] and this increase is higher if animals are fed processed DG as opposed to unprocessed grain [106] (Figure 2). 

Generic *E. coli* (STEC and non-STEC) has been reported to increase during grain-induced SARA [6,155,156,157]. On the basis of this finding, it was hypothesized that acidic conditions may promote the emergence of acid resistant STEC [158]. However, the comparison between forage- and grain-fed animals showed that low acidity in the rumen is unrelated to the development of acid resistance, as passage through the rumen is sufficient to turn acid sensitive to acid resistant (AR) STEC [158]. The mechanism that STEC uses to become AR relies upon the activation of the GAD pathway through the AHL compounds presence in the rumen [121]. STEC cannot produce but it can detect AHL signals from different rumen symbionts through the SdiA protein, and upon detection of these signals, the LEE gene expression shuts down, while that of the *gad* genes is upregulated [121]. The SdiA system regulation is functional in both grain and forage diets, even though the GAD activation appears to be more important for the survival of STEC in grain-fed animals [122]. 

### 4.3. Other Husbandry Practices Related to STEC Emergence

Fasting, which often occurs during and after the transport of animals, has also been associated with increased pH, decreased SCFA and increased O157:H7 abundance [48]. The administration of antimicrobials, typically added to cattle diet to improve animal growth and weight gain [4], a practice that has been banned in Europe (IP/05/1687), can intensify the *E. coli* O157:H7 threat. Monensin, an antimicrobial commonly used in the US, has no or little effect on gram-negative bacteria (e.g., *E. coli*), and therefore, administration has either no effect on *E. coli* O157:H7 [19,159] or causes an increase in *E. coli* O157:H7 shedding [4]. In studies where monensin administration resulted in a reduction in *E. coli* O157:H7 shedding, the animals received the highest possible concentration (44mg/kg), which possibly resulted in the elimination of beneficial symbionts and reduced fermentation in the hindgut [160].

Animal husbandry practices can further favor *E. coli* O157:H7 transmission in a cumulative manner: when animals in crowded farms are fed high grain, prevalence of *E. coli* O157:H7 increases as super-shedders can transmit O157:H7 to other animals in the same pen [161]. Within farms, feed and water contamination have been suggested to promote *E. coli* O157:H7 transmission. However, fecal contamination of pen floors and animal hides poses an even greater risk for *E. coli* O157:H7 transmission [19]. In a study that examined the potential connection between farm conditions and *E. coli* O157:H7 prevalence, it was demonstrated that muddy pens increase the chance of shedding [162]. Flies that belong to the *Muscidae* and *Calliphoridae* families facilitate *E. coli* O157:H7 transmission to cattle [19] (Figure 1), to vegetation [163] and possibly humans [19]. Finally, animal stress results in the release of norepinephrine in the cattle GIT, which promotes *E. coli* O157:H7 growth [19].

## 5. STEC Prevention Methods and Future Perspectives

Strategies to control STEC in cattle have so far focused on eliminating STEC in the animal GIT, feces and farm environment (i.e., feed, pen floors). These strategies aim to: (i) limit the occurrence of STEC in meat and dairy products and prevent it from entering the food chain and (ii) reduce the financial losses from products that are discarded as a result of STEC contamination [164]. Most strategies typically aim to reduce the STEC load in cattle, but regular cleaning of feedlots and pen surfaces and provision of dry bedding have also been proposed as methods to reduce STEC prevalence. 

### 5.1. Active and Passive Vaccination

Active (through intramuscular injection) and passive (through colostrum administration) vaccination have been proposed as methods to control STEC. Colostrum administration to newborn calves facilitates the transfer of maternal antibodies (Abs) that protect until the calf develops its own immune responses [110]. Thus, if dams have a significant humoral immune response against STEC, then colostrum administration should facilitate the vertical transmission of STEC-specific Abs, which would result in reduced STEC shedding. However, even though Ab transfer from (vaccinated) dams to their calves through colostrum administration has been demonstrated [165,166,167], shedding is not always reduced [168]. In addition, results are often hard to interpret [169], as colostrum administration from multiple dams to calves is more efficient in reducing STEC shedding than colostrum administration from their own mother [170]. This would suggest that transmission of maternal Abs may not be the (only) reason colostrum administration reduces STEC shedding, but it may be related to other beneficial effects triggered by colostrum administration, such as the faster or more efficient establishment of GIT microbial symbionts [110]. 

STEC rarely causes symptoms (except occasional diarrhea in newborn calves) and appears to be immunotolerated in cattle [45,171,172]. It has been proposed by Schaut and colleagues [173] that this could be part of an interplay between STEC and host, similar to the one observed in humans between *E. coli* symbionts and the host. STEC immunotolerance in cattle has also been attributed to low STEC abundance [171], the inability of Stx to reach the site that initiates an immune response [45] or a potential immunosuppression/immunodelay caused by Stx [172]. On the basis of these previous works, immunization with inactivated forms of Shiga toxin was suggested and trials have demonstrated somewhat promising results [173,174]. Active vaccination targeting STEC Type III secreted proteins (e.g, Esps, Tir) has demonstrated a reduction in *E. coli* O157:H7 shedding [175] although the effect is not consistent [176] and varies among animals. Therefore, even though vaccination holds significant theoretical promise that has led to field trials and commercialization of anti-EHEC vaccines (Canada), the tests were not sufficiently encouraging to result in widespread use [177]. 

### 5.2. Bacteriophages

The use of bacteriophages, typically isolated from cattle feces, against *E. coli* O157:H7 and non-O157 has reported promising results. However, the evaluation of their efficiency can be challenging, as the presence of other phages in the cattle GIT can make interpretation of results difficult [178]. STEC control using bacteriophages against STEC contaminated surfaces in cattle farms (e.g., cow hides) and meat products has demonstrated a significant decrease in STEC [114,139]. However, even though in vitro assays have shown promising results [137,138,139], in vivo trials have not always been successful. Administration of bacteriophages can result in STEC reduction, although in some studies, the reduction between treatment groups was not significant and in some other studies, there was no reduction at all [114]. 

### 5.3. Non-Microbial Feed Additives

Various feed and water additives, such as orange, citrus peel and pulp and tannin extracts, have reported positive results in controlling *E. coli* O157:H7 in cattle [48]. Lactoferrin, an iron-binding glycoprotein that exhibits antibacterial activity, can eliminate *E. coli* O157:H7 shedding if administered by rectal injection but not orally [179]. However, most of these results originate from small trials and single studies, and therefore, extensive testing is required to verify their potential anti-O157:H7 properties and examine their impact on the rest of the microbiota (since many antimicrobials are wide range) [19,48]. 

### 5.4. Direct-Fed Microbials

DFMs can effectively reduce the *E. coli* O157 shedding in cattle [21] and the summarized beneficial modes of action that are likely responsible are: (i) competitive exclusion, (ii) production of antimicrobial compounds, (iii) reduced risk for ruminal acidosis [99] and (iv) potential beneficial interactions with members of the GIT microbiota. LAB strains isolated from cattle feces have shown promising results against *E. coli* O157 through lactic acid production and pH reduction [113,158]. The administration of combined *Lactobacillus acidophilus* and *Propionibacterium freudenreichii* against *E. coli* O157 shedding has been widely tested and showed better efficacy than other DFMs [18,21]. Combined *Streptococcus bovis* and *Lactobacillus gallinarum* have also reported promising results [21,180]. In one of the first studies that used DFMs against *E. coli* O157:H7, the reduced shedding was attributed to an increase in SCFA in feces, which was suggested to have bactericidal (anti-gram negative) activity [180]. In a recent study, Bertin and colleagues [20] demonstrated that *L. reuteri* catabolizes glycerol to produce HPA, which inhibits *E. coli* O157:H7 in vitro. Administration of DFMs also has an important role in early life cattle; if *Faecalibacterium prausnitzii* is administered early in life, it can reduce the incidence of diarrhea (caused by *E. coli* pathotypes) as it persists within the digestive tract for several weeks [110]. 

### 5.5. Towards New Strategies for STEC Control

Considering the effectiveness of DFMs against STEC colonization in cattle, the rich diversity of cattle GIT microbiota and the low abundance of STEC in the GIT, it is very likely that the GIT microbiota offers a promising field for the discovery of next generation DFMs. Several lines of indirect evidence support this hypothesis; even though STEC is very prevalent in the cattle farm environment [50,51,52] and is easily transmitted among animal hosts [53,54], its prevalence and abundance in cattle is never high (see above). This suggests that there are host or other factors preventing its colonization. Since healthy cattle carry STEC asymptomatically and STEC carriage does not affect cattle growth or reproduction, natural (host) selection for defenses against STEC colonization is “invisible”. However, natural selection against STEC could be triggered by conflict with the native GIT microbiota, possibly a result of competition between gut symbionts and STEC for resources. 

The existence of STEC antagonists within the cattle microbiome has further been suggested by 16S metagenomic studies that compared the rectal microbiota of super-shedders (SS) and non-shedders (NS) in order to identify potential patterns of mutually exclusivity or co-occurrence between *E. coli* O157:H7 and members of the microbiota [116,117,118,119,120]. These studies took place explicitly in the US and Canada and mainly focused on steers fed diets that included grain (Table 1). Even though comparisons of rectal bacterial communities in SS and NS did not demonstrate sharp contrasts in ɑ- or β-diversity, all studies identified some taxa (at phylum, family or genus level) with a preference towards SS or NS. Members of Firmicutes (order Clostridiales) and Bacteroidetes (order Bacteroidales), which are also the most prevalent phyla in the cattle GIT (Figure 2), are the most commonly differentially represented taxa in SS and NS animals (Table 1). Bacterial taxa belonging to *Ruminococcaceae*, *Paraprevotellaceae* or *Alcaligenaceae* are present more in NS. Taxa within *Prevotellaceae*, *Clostridiaceae, Erysipelotrichaceae*, *Lachnospiraceae* and *Ruminococcaceae* appear overrepresented in both NS and SS (Table 1). Although pioneering, these correlative studies need to be followed up by shotgun metagenomics/whole genome sequencing and comparative genomics, which will allow us to better identify potential STEC antagonists and examine their genomic potential. Such insights can then be followed up (whenever possible) by in vitro experiments. The search for STEC antagonists in the cattle microbiota has the potential to identify specialized gut symbionts that have adapted to an endosymbiotic lifestyle and possibly coevolved with the cattle host. Such symbionts are likely to provide mutualistic services to the host (nutritional and other)—in addition to preventing STEC colonization—and therefore, this type of research could identify DFMs that will not only eliminate STEC but may also improve the wellbeing and productivity of ruminants. 

## Figures and Tables

**Table 1:** Summary of 16S comparative metagenomic studies comparing (NS) non-shedders and *E. coli* O157:H7 super-shedders (SS). **A**: Statistically significant differentially abundant taxa between NS and SS (based on the statistical analyses of the cited studies) and the corresponding references are presented. **B**: Additional information about the experimental design of the original studies.

A					
NS			SS		
Phylum	Family/Order	Authors	Phylum	Family/Order	Authors
Actinobacteria	*Intrasporangiaceae*	Wang et al. [116]	Actinobacteria	*Actinomycetaceae*	Salaheen et al. [117]
Actinobacteria	*Thermomonosporaceae*	Salaheen et al. [117]	Actinobacteria	*Corynebacteriaceae*	Wang et al. [116]
Bacteroidetes	*Paraprevotellaceae*	Wang et al., Zaheer et al. [116,118]	Actinobacteria	*Gordoniaceae*	Wang et al. [116]
Bacteroidetes	*Prevotellaceae*	Zaheer et al. [118]	Actinobacteria	*Propionibacteriaceae*	Wang et al. [116]
Bacteroidetes	*Sphingobacteriaceae*	Wang et al. [116]	Actinobacteria	*Unclassified Bifidobacteriales*	Salaheen et al. [117]
Cyanobacteria	*Cyanophyceae*	Salaheen et al. [117]	Bacteroidetes	*Marinilabiliaceae*	Salaheen et al. [117]
Cyanobacteria	*Synechococcaceae*	Salaheen et al. [117]	Bacteroidetes	*Prevotellaceae*	Xu et al. [119]
Firmicutes	*Bacillaceae*	Stenkamp-Strahm et al. [120]	Bacteroidetes	*Rikenellaceae*	Zaheer et al., Xu et al. [118,119]
Firmicutes	*Clostridiaceae*	Xu et al., Salaheen et al. [117,119]	Bacteroidetes	*Sphingobacteriaceae*	Salaheen et al. [117]
Firmicutes	*Erysipelotrichaceae*	Xu et al. [119]	Firmicutes	*Clostridiaceae*	Xu et al. [119]
Firmicutes	*Heliobacteriaceae*	Salaheen et al. [117]	Firmicutes	*Erysipelotrichaceae*	Wang et al. [116]
Firmicutes	*Lachnospiraceae*	Wang et al., Xu et al., Salaheen et al. [116,117,119]	Firmicutes	*Lachnospiraceae*	Stenkamp-Strahm, Xu et al. [119,120]
Firmicutes	*Ruminococcaceae*	Wang et al., Zaheer et al., Xu et al. [116,118,119]	Firmicutes	*Lactobacillaceae*	Wang et al. [116]
Firmicutes	*Sporolactobacillaceae*	Salaheen et al. [117]	Firmicutes	*Mogibacteriaceae*	Wang et al. [116]
Firmicutes	*Streptococcaceae*	Salaheen et al. [117]	Firmicutes	*Peptococcaceae*	Xu et al. [119]
Firmicutes	*Veillonellaceae*	Wang et al. [116]	Firmicutes	*Ruminococcaceae*	Zaheer et al., Xu et al. [118,119]
Fusobacteria	*Fusobacteriaceae*	Wang et al. [116]	Firmicutes	*Staphylococcaceae*	Wang et al. [116]
Proteobacteria	*Alcaligenaceae*	Wang et al., Zaheer et al. [116,118]	Firmicutes	*Veillonellaceae*	Wang et al. [116]
Proteobacteria	*Bdellovibrionaceae*	Wang et al. [116]	Proteobacteria	*Moraxellaceae*	Wang et al. [116]
Proteobacteria	*Bradyrhizobiaceae*	Wang et al. [116]	Proteobacteria	Rhodobacterales	Wang et al. [116]
Proteobacteria	*Comamonadaceae*	Salaheen et al. [117]			
Proteobacteria	*Desulfovibrionaceae*	Xu et al. [119]			
Proteobacteria	*Nannocystaceae*	Salaheen et al. [117]			
Proteobacteria	*Rhodospirillaceae*	Salaheen et al. [117]			
Proteobacteria	*Shewanellaceae*	Wang et al. [116]			
Proteobacteria	*Succinivibrionaceae*	Zaheer et al. [118]			
Spirochaetes	*Spirochaetaceae*	Zaheer et al. [118]			
Spirochaetes	Unclassified *Leptospiraceae*	Salaheen et al. [117]			
Spirochaetes	Unclassified Spirochaetales	Salaheen et al. [117]			
Verrucomicrobia	Unclassified Chthoniobacterales	Salaheen et al. [117]			
**B**					
**doi Number**		**First Author**	**Tissue**	**Country**	**Animal**
10.1128/AEM.01738-17 [116]		Wang	rectum	Canada	beef steers
10.1111/jam.13679 [120]		Stenkamp-Strahm	rectum	US	Holstein-Friesian cows
10.1371/journal.pone.0170050 [118]		Zaheer	lower GI tract	Canada	beef steers
10.1371/journal.pone.0098115 [119]		Xu	rectum	Canada	feedlot steers
10.1016/j.foodcont.2019.03.022 [117]		Salaheen	feces	US	lactating cows

## References

[1] Diamond J. (2002). Evolution, consequences and future of plant and animal domestication. Nature.

[2] Hobson P.N., Stewart C.S. (1997). The Rumen Microbial Ecosystem.

[3] Schingoethe D.J., Kalscheur K.F., Hippen A.R., Garcia A.D. (2009). Invited review: The use of distillers products in dairy cattle diets. J. Dairy Sci..

[4] Hales K.E., Wells J.E., Berry E.D., Kalchayanand N., Bono J.L., Kim M. (2017). The effects of monensin in diets fed to finishing beef steers and heifers on growth performance and fecal shedding of *Escherichia coli* O157:H7. J. Anim. Sci..

[5] Gilchrist M.J., Greko C., Wallinga D.B., Beran G.W., Riley D.G., Thorne P.S. (2007). The potential role of concentrated animal feeding operations in infectious disease epidemics and antibiotic resistance. Environ. Health Perspect..

[6] Diez-Gonzalez F., Callaway T.R., Kizoulis M.G., Russell J.B. (1998). Grain feeding and the dissemination of acid-resistant *Escherichia coli* from cattle. Science.

[7] Karmali M.A., Mascarenhas M., Shen S., Ziebell K., Johnson S., Reid-Smith R., Isaac-Renton J., Clark C., Rahn K., Kaper J.B. (2003). Association of genomic O island 122 of *Escherichia coli* EDL 933 with verocytotoxin-producing *Escherichia coli* seropathotypes that are linked to epidemic and/or serious disease. J. Clin. Microbiol..

[8] Laing C.R., Buchanan C., Taboada E.N., Zhang Y., Karmali M.A., Thomas J.E., Gannon V.P. (2009). In silico genomic analyses reveal three distinct lineages *of Escherichia coli* O157:H7, one of which is associated with hyper-virulence. BMC Genom..

[9] Koutsoumanis K., Allende A., Alvarez-Ordóñez A., Bover-Cid S., Chemaly M., Davies R., Cesare A.D., Herman L., Hilbert F., Lindqvist R. (2020). Pathogenicity assessment of Shiga toxin-producing *Escherichia coli* (STEC) and the public health risk posed by contamination of food with STEC. EFSA J..

[10] Caprioli A., Morabito S., Brugère H., Oswald E. (2005). Enterohaemorrhagic *Escherichia coli*: Emerging issues on virulence and modes of transmission. Vet. Res..

[11] Conrad C.C., Stanford K., Narvaez-Bravo C., Callaway T., McAllister T. (2016). Farm fairs and petting zoos: A review of animal contact as a source of zoonotic enteric disease. Foodborne Pathog. Dis..

[12] Ferens W.A., Hovde C.J. (2011). Escherichia coli O157:H7: Animal reservoir and sources of human infection. Foodborne Pathog. Dis..

[13] Brugère H., Auvray F., Mariani-Kurkdjian P., King L.A., Loukiadis E. (2012). *E coli* producteurs de shigatoxines (STEC): Définitions, virulence et propriétés des souches entérohémorragiques (EHEC). Fr. Épidémiologique St. Anim. Aliment..

[14] Burger R. (2012). EHEC O104:h4 in germany 2011: Large Outbreak of Bloody Diarrhea and Haemolytic Uraemic Syndrome by Shiga Toxin–Producing E. coli Via Contaminated Food.

[15] Fatima R., Aziz M. (2019). Enterohemorrhagic *Escherichia coli* (EHEC). StatPearls.

[16] Chase-Topping M., Gally D., Low C., Matthews L., Woolhouse M. (2008). Super-shedding and the link between human infection and livestock carriage of escherichia coli O157. Nat. Rev. Microbiol..

[17] Duffy G., McCabe E. (2014). Veterinary public health approach to managing pathogenic verocytotoxigenic *Escherichia coli* in the agri-food Chain. Microbiol. Spectr..

[18] Chaucheyras-Durand F., Dunière L., Forano E. (2016). Comment garantir la sécurité microbiologique de la viande bovine ?. Rev. Viandes Prod. Carnés Artic. Sci..

[19] Berry E.D., Wells J.E., Taylor S.L. (2010). Chapter 4—*Escherichia coli* O157:H7: Recent advances in research on occurrence, transmission, and control in cattle and the production environment. Advances in Food and Nutrition Research.

[20] Bertin Y., Habouzit C., Dunière L., Laurier M., Durand A., Duchez D., Segura A., Thévenot-Sergentet D., Baruzzi F., Chaucheyras-Durand F. (2017). *Lactobacillus reuteri* suppresses *E. coli* O157:H7 in bovine ruminal fluid: Toward a pre-slaughter strategy to improve food safety?. PLoS ONE.

[21] Wisener L.V., Sargeant J.M., O’Connor A.M., Faires M.C., Glass-Kaastra S.K. (2015). The use of direct-fed microbials to reduce shedding of *Escherichia coli* O157 in beef cattle: A systematic review and meta-analysis. Zoonoses Public Health.

[22] McAllister T.A., Beauchemin K.A., Alazzeh A.Y., Baah J., Teather R.M., Stanford K. (2011). Review: The use of direct fed microbials to mitigate pathogens and enhance production in cattle. Can. J. Anim. Sci..

[23] Uyeno Y., Shigemori S., Shimosato T. (2015). Effect of probiotics/prebiotics on cattle health and productivity. Microbes Environ..

[24] Gomes T.A.T., Elias W.P., Scaletsky I.C.A., Guth B.E.C., Rodrigues J.F., Piazza R.M.F., Ferreira L.C.S., Martinez M.B. (2016). Diarrheagenic. Escherichia Coli. Braz. J. Microbiol..

[25] Elder J., Nightingale K., Sofos J. (2013). 12—Tracking of pathogens via virulence factors: Shiga toxin-producing *Escherichia coli* in cattle and potential risks for human disease. Advances in Microbial Food Safety.

[26] Kaper J.B., Nataro J.P., Mobley H.L. (2004). Pathogenic *Escherichia coli*. Nat. Rev. Microbiol..

[27] O’Loughlin E.V., Robins-Browne R.M. (2001). Effect of Shiga toxin and Shiga-like toxins on eukaryotic cells. Microbes Infect..

[28] Siegler R.L. (2003). Postdiarrheal Shiga toxin-mediated hemolytic uremic syndrome. JAMA.

[29] Bruyand M., Mariani-Kurkdjian P., Hello S.L., Lefevre S., Jourdan-Da Silva N., Nisavanh A., Mailles A., Bonacorsi S., De Valk H. (2017). Surveillance du Syndrome Hémolytique et Urémique Post-Diarrhéique Chez L’enfant de Moins de 15 Ans en France en 2017.

[30] Van Elsas J.D., Semenov A.V., Costa R., Trevors J.T. (2011). Survival of *Escherichia coli* in the environment: Fundamental and public health aspects. ISME J..

[31] Gyles C.L. (2007). Shiga toxin-producing *Escherichia coli*: An overview. J. Anim. Sci..

[32] Mathusa E.C., Chen Y., Enache E., Hontz L. (2010). Non-O157 Shiga toxin-producing *Escherichia coli* in foods. J. Food Prot..

[33] Amézquita-López B.A., Soto-Beltrán M., Lee B.G., Yambao J.C., Quiñones B. (2018). Isolation, genotyping and antimicrobial resistance of Shiga toxin-producing *Escherichia coli*. J. Microbiol. Immunol. Infect..

[34] Nataro J.P., Kaper J.B. (1998). Diarrheagenic *Escherichia coli*. Clin. Microbiol. Rev..

[35] Dean-Nystrom E.A., Bosworth B.T., Cray W.C., Moon H.W. (1997). Pathogenicity of *Escherichia coli* O157:H7 in the intestines of neonatal calves. Infect. Immun..

[36] Scheutz F., Teel L.D., Beutin L., Piérard D., Buvens G., Karch H., Mellmann A., Caprioli A., Tozzoli R., Morabito S. (2012). Multicenter evaluation of a sequence-based protocol for subtyping Shiga toxins and standardizing Stx nomenclature. J. Clin. Microbiol..

[37] Pacheco A.R., Sperandio V. (2012). Shiga toxin in enterohemorrhagic *E. coli*: Regulation and novel anti-virulence strategies. Front. Cell. Infect. Microbiol..

[38] Coombes B.K., Wickham M.E., Mascarenhas M., Gruenheid S., Finlay B.B., Karmali M.A. (2008). Molecular Analysis as an Aid To Assess the Public Health Risk of Non-O157 Shiga Toxin-Producing *Escherichia coli* Strains. Appl. Environ. Microbiol..

[39] Nielsen E.M., Skov M.N., Madsen J.J., Lodal J., Jespersen J.B., Baggesen D.L. (2004). Verocytotoxin-Producing *Escherichia coli* in Wild Birds and Rodents in Close Proximity to Farms. Appl. Environ. Microbiol..

[40] Le Gall T., Clermont O., Gouriou S., Picard B., Nassif X., Denamur E., Tenaillon O. (2007). Extraintestinal virulence is a coincidental by-product of commensalism in B2 phylogenetic group *Escherichia coli* strains. Mol. Biol. Evol..

[41] Leimbach A., Hacker J., Dobrindt U.E. (2013). coli as an all-rounder: The thin line between commensalism and pathogenicity. Curr. Top. Microbiol. Immunol..

[42] Diard M., Garry L., Selva M., Mosser T., Denamur E., Matic I. (2010). Pathogenicity-associated islands in extraintestinal pathogenic *Escherichia coli* are fitness elements involved in intestinal colonization. J. Bacteriol..

[43] Dean-Nystrom E.A., Bosworth B.T., Moon H.W., O’Brien A.D. (1998). *Escherichia coli* O157:H7 Requires Intimin for Enteropathogenicity in Calves. Infect. Immun..

[44] Smith D.G.E., Naylor S.W., Gally D.L. (2002). Consequences of EHEC colonisation in humans and cattle. Int. J. Med. Microbiol. IJMM.

[45] Fröhlich J., Baljer G., Menge C. (2009). Maternally and naturally acquired antibodies to Shiga Toxins in a cohort of calves shedding Shiga-Toxigenic *Escherichia coli*. Appl. Environ. Microbiol..

[46] Meltz Steinberg K., Levin B.R. (2007). Grazing protozoa and the evolution of the *Escherichia coli* O157:H7 Shiga toxin-encoding prophage. Proc. R. Soc. B Biol. Sci..

[47] Schmidt C.E., Shringi S., Besser T.E. (2016). Protozoan Predation of *Escherichia coli* O157:H7 Is Unaffected by the Carriage of Shiga Toxin-Encoding Bacteriophages. PLoS ONE.

[48] Callaway T.R., Carr M.A., Edrington T.S., Anderson R.C., Nisbet D.J. (2009). Diet, *Escherichia coli* O157:H7, and cattle: A review after 10 years. Curr. Issues Mol. Biol..

[49] Wells J.E., Kim M., Bono J.L., Kuehn L.A., Benson A.K. (2014). Meat science and muscle biology symposium: *Escherichia coli* O157:H7, diet, and fecal microbiome in beef cattle. J. Anim. Sci..

[50] Wang G., Zhao T., Doyle M.P. (1996). Fate of enterohemorrhagic *Escherichia coli* O157:H7 in bovine feces. Appl. Environ. Microbiol..

[51] Fukushima H., Hoshina K., Gomyoda M. (1999). Long-term survival of Shiga toxin-producing *Escherichia coli* O26, O111, and O157 in Bovine Feces. Appl. Environ. Microbiol..

[52] Fremaux B., Prigent-Combaret C., Vernozy-Rozand C. (2008). Long-term survival of Shiga toxin-producing *Escherichia coli* in cattle effluents and environment: An updated review. Vet. Microbiol..

[53] Besser T.E., Richards B.L., Rice D.H., Hancock D.D. (2001). *Escherichia coli* O157:H7 infection of calves: Infectious dose and direct contact transmission. Epidemiol. Infect..

[54] Semenov A.M., Kuprianov A.A., Van Bruggen A.H.C. (2010). Transfer of enteric pathogens to successive habitats as part of microbial cycles. Microb. Ecol..

[55] Shaw D.J., Jenkins C., Pearce M.C., Cheasty T., Gunn G.J., Dougan G., Smith H.R., Woolhouse M.E.J., Frankel G. (2004). Shedding patterns of verocytotoxin-producing *Escherichia coli* strains in a cohort of calves and their dams on a scottish beef farm. Appl. Environ. Microbiol..

[56] Islam M.Z., Musekiwa A., Islam K., Ahmed S., Chowdhury S., Ahad A., Biswas P.K. (2014). Regional variation in the prevalence of *E. coli* O157 in cattle: A meta-analysis and meta-regression. PLoS ONE.

[57] Bibbal D., Loukiadis E., Kérourédan M., Ferré F., Dilasser F., De Peytavin Garam C., Cartier P., Oswald E., Gay E., Auvray F. (2015). Prevalence of carriage of Shiga toxin-producing *Escherichia coli* serotypes O157:H7, O26:H11, O103:H2, O111:H8, and O145:H28 among slaughtered adult cattle in France. Appl. Environ. Microbiol..

[58] Ekong P., Sanderson M., Cernicchiaro N. (2015). Prevalence and concentration of *Escherichia coli* O157 in different seasons and cattle types processed in North America: A systematic review and meta-analysis of published research. Prev. Vet. Med..

[59] Nielsen E.M., Tegtmeier C., Andersen H.J., Grønbaek C., Andersen J.S. (2002). Influence of age, sex and herd characteristics on the occurrence of Verocytotoxin-producing *Escherichia coli* O157 in Danish dairy farms. Vet. Microbiol..

[60] Mir R.A., Weppelmann T.A., Kang M., Bliss T.M., DiLorenzo N., Lamb G.C., Ahn S., Jeong K.C. (2015). Association between animal age and the prevalence of Shiga toxin-producing *Escherichia coli* in a cohort of beef cattle. Vet. Microbiol..

[61] Zhao L., Tyler P.J., Starnes J., Bratcher C.L., Rankins D., McCaskey T.A., Wang L. (2013). Correlation analysis of Shiga toxin-producing *Escherichia coli* shedding and faecal bacterial composition in beef cattle. J. Appl. Microbiol..

[62] Stanford K., Johnson R.P., Alexander T.W., McAllister T.A., Reuter T. (2016). Influence of season and feedlot location on prevalence and virulence factors of seven serogroups of *Escherichia coli* in feces of Western-Canadian slaughter cattle. PLoS ONE.

[63] Li H., Li R., Chen H., Gao J., Wang Y., Zhang Y., Qi Z. (2020). Effect of different seasons (spring vs. summer) on the microbiota diversity in the feces of dairy cows. Int. J. Biometeorol..

[64] Grauke L.J., Kudva I.T., Yoon J.W., Hunt C.W., Williams C.J., Hovde C.J. (2002). Gastrointestinal tract location of *Escherichia coli* O157:H7 in ruminants. Appl. Environ. Microbiol..

[65] Keen J.E., Laegreid W.W., Chitko-McKown C.G., Durso L.M., Bono J.L. (2010). Distribution of Shiga-toxigenic *Escherichia coli* O157 in the gastrointestinal tract of naturally O157-shedding cattle at necropsy. Appl. Environ. Microbiol..

[66] Naylor S.W., Low J.C., Besser T.E., Mahajan A., Gunn G.J., Pearce M.C., McKendrick I.J., Smith D.G.E., Gally D.L. (2003). Lymphoid follicle-dense mucosa at the terminal rectum is the principal site of colonization of enterohemorrhagic *Escherichia coli* O157:H7 in the bovine host. Infect. Immun..

[67] Boukhors K., Pradel N., Girardeau J.-P., Livrelli V., Ou Saïd A.M., Contrepois M., Martin C. (2002). Effect of diet on Shiga toxin-producing *Escherichia coli* (STEC) growth and survival in rumen and abomasum fluids. Vet. Res..

[68] Kudva I.T., Stanton T.B., Lippolis J.D. (2014). The *Escherichia coli* O157:H7 bovine rumen fluid proteome reflects adaptive bacterial responses. BMC Microbiol..

[69] Lim J.Y., Sheng H., Seo K.S., Park Y.H., Hovde C.J. (2007). Characterization of an *Escherichia coli* O157:H7 plasmid O157 deletion mutant and its survival and persistence in cattle. Appl. Environ. Microbiol..

[70] Price S.B., Wright J.C., DeGraves F.J., Castanie-Cornet M.-P., Foster J.W. (2004). Acid resistance systems required for survival of *Escherichia coli* O157:H7 in the bovine gastrointestinal tract and in apple cider are different. Appl. Environ. Microbiol..

[71] Free A.L., Duoss H.A., Bergeron L.V., Shields-Menard S.A., Ward E., Callaway T.R., Carroll J.A., Schmidt T.B., Donaldson J.R. (2012). Survival of O157:H7 and Non-O157 Serogroups of *Escherichia coli* in Bovine Rumen Fluid and Bile Salts. Foodborne Pathog. Dis..

[72] Hamner S., McInnerney K., Williamson K., Franklin M.J., Ford T.E. (2013). Bile salts affect expression of *Escherichia coli* O157:H7 genes for virulence and iron acquisition, and promote growth under iron limiting conditions. PLoS ONE.

[73] Moran J. (2005). How the rumen works. Tropical Dairy Farming: Feeding Management for Small Holder Dairy Farmers in the Humid Tropics.

[74] Monteiro R., Ageorges V., Rojas-Lopez M., Schmidt H., Weiss A., Bertin Y., Forano E., Jubelin G., Henderson I.R., Livrelli V. (2016). A secretome view of colonisation factors in Shiga toxin-encoding *Escherichia coli* (STEC): From enterohaemorrhagic *E. coli* (EHEC) to related enteropathotypes. FEMS Microbiol. Lett..

[75] McWilliams B.D., Torres A.G. (2014). Enterohemorrhagic *Escherichia coli* adhesins. Microbiol. Spectr..

[76] Ageorges V., Monteiro R., Leroy S., Burgess C.M., Pizza M., Chaucheyras-durand F., Desvaux M. (2020). Molecular determinants of surface colonisation in diarrhoeagenic *Escherichia coli* (DEC): From bacterial adhesion to biofilm formation. FEMS Microbiol. Rev..

[77] Dziva F., Van Diemen P.M., Stevens M.P., Smith A.J., Wallis T.S. (2004). Identification of *Escherichia coli* O157: H7 genes influencing colonization of the bovine gastrointestinal tract using signature-tagged mutagenesis. Microbiol. Read. Engl..

[78] Naylor S.W., Roe A.J., Nart P., Spears K., Smith D.G.E., Low J.C., Gally D.L. (2005). *Escherichia coli* O157: H7 forms attaching and effacing lesions at the terminal rectum of cattle and colonization requires the LEE4 operon. Microbiology.

[79] Baehler A.A., Moxley R.A. (2000). Escherichia coli O157:H7 induces attaching-effacing lesions in large intestinal mucosal explants from adult cattle. FEMS Microbiol. Lett..

[80] Phillips A., Navabpour S., Hicks S., Dougan G., Wallis T., Frankel G. (2000). Enterohaemorrhagic *Escherichia coli* O157:H7 target Peyer’s patches in humans and cause attaching/effacing lesions in both human and bovine intestine. GUT.

[81] Low J.C., McKendrick I.J., McKechnie C., Fenlon D., Naylor S.W., Currie C., Smith D.G.E., Allison L., Gally D.L. (2005). Rectal carriage of enterohemorrhagic *Escherichia coli* O157 in slaughtered cattle. Appl. Environ. Microbiol..

[82] Sharma V.K., Sacco R.E., Kunkle R.A., Bearson S.M.D., Palmquist D.E. (2012). Correlating levels of type III secretion and secreted proteins with fecal shedding of *Escherichia coli* O157:H7 in cattle. Infect. Immun..

[83] Van Diemen P.M., Dziva F., Stevens M.P., Wallis T.S. (2005). Identification of enterohemorrhagic *Escherichia coli* O26:H−Genes required for intestinal colonization in calves. Infect. Immun..

[84] Mahajan A., Currie C.G., Mackie S., Tree J., McAteer S., McKendrick I., McNeilly T.N., Roe A., La Ragione R.M., Woodward M.J. (2009). An investigation of the expression and adhesin function of H7 flagella in the interaction of *Escherichia coli* O157: H7 with bovine intestinal epithelium. Cell. Microbiol..

[85] Erdem A.L., Avelino F., Xicohtencatl-Cortes J., Girón J.A. (2007). Host protein binding and adhesive properties of H6 and H7 flagella of attaching and effacing *Escherichia coli*. J. Bacteriol..

[86] Low A.S., Dziva F., Torres A.G., Martinez J.L., Rosser T., Naylor S., Spears K., Holden N., Mahajan A., Findlay J. (2006). Cloning, expression, and characterization of fimbrial operon F9 from enterohemorrhagic *Escherichia coli* O157:H7. Infect. Immun..

[87] Kudva I.T., Griffin R.W., Krastins B., Sarracino D.A., Calderwood S.B., John M. (2012). Proteins other than the locus of enterocyte effacement-encoded proteins contribute to *Escherichia coli* O157:H7 adherence to bovine rectoanal junction stratified squamous epithelial cells. BMC Microbiol..

[88] Xicohtencatl-Cortes J., Monteiro-Neto V., Ledesma M.A., Jordan D.M., Francetic O., Kaper J.B., Puente J.L., Girón J.A. (2007). Intestinal adherence associated with type IV pili of enterohemorrhagic *Escherichia coli* O157:H7. J. Clin. Investig..

[89] Ooka T., Seto K., Kawano K., Kobayashi H., Etoh Y., Ichihara S., Kaneko A., Isobe J., Yamaguchi K., Horikawa K. (2012). Clinical significance of *Escherichia albertii*. Emerg. Infect. Dis..

[90] Fitzhenry R.J., Pickard D.J., Hartland E.L., Reece S., Dougan G., Phillips A.D., Frankel G. (2002). Intimin type influences the site of human intestinal mucosal colonisation by enterohaemorrhagic *Escherichia coli* O157:H7. GUT.

[91] Koike S., Kobayashi Y. (2009). Fibrolytic rumen bacteria: Their ecology and functions. Asian Aust. J. Anim. Sci..

[92] Dodd D., Mackie R.I., Cann I.K.O. (2011). Xylan degradation, a metabolic property shared by rumen and human colonic Bacteroidetes. Mol. Microbiol..

[93] Seshadri R., Leahy S.C., Attwood G.T., Teh K.H., Lambie S.C., Cookson A.L., Eloe-Fadrosh E.A., Pavlopoulos G.A., Hadjithomas M., Varghese N.J. (2018). Cultivation and sequencing of rumen microbiome members from the Hungate1000 Collection. Nat. Biotechnol..

[94] Stewart R.D., Auffret M.D., Warr A., Wiser A.H., Press M.O., Langford K.W., Liachko I., Snelling T.J., Dewhurst R.J., Walker A.W. (2018). Assembly of 913 microbial genomes from metagenomic sequencing of the cow rumen. Nat. Commun..

[95] Wallace R.J., Snelling T.J., McCartney C.A., Tapio I., Strozzi F. (2017). Application of meta-omics techniques to understand greenhouse gas emissions originating from ruminal metabolism. Genet. Sel. Evol. GSE.

[96] Stewart C.S., Wood B.J.B. (1992). Lactic acid bacteria in the rumen. The Lactic Acid Bacteria Volume 1: The Lactic Acid Bacteria in Health and Disease.

[97] Yang H.E., Zotti C.A., McKinnon J.J., McAllister T.A. (2018). Lactobacilli are prominent members of the microbiota involved in the ruminal digestion of barley and corn. Front. Microbiol..

[98] Huws S.A., Creevey C.J., Oyama L.B., Mizrahi I., Denman S.E., Popova M., Muñoz-Tamayo R., Forano E., Waters S.M., Hess M. (2018). Addressing global ruminant agricultural challenges through understanding the rumen microbiome: Past, present, and future. Front. Microbiol..

[99] Plaizier J.C., Danesh Mesgaran M., Derakhshani H., Golder H., Khafipour E., Kleen J.L., Lean I., Loor J., Penner G., Zebeli Q. (2018). Review: Enhancing gastrointestinal health in dairy cows. Anim. Int. J. Anim. Biosci..

[100] Kameshwar A.K.S., Ramos L.P., Qin W. (2019). Metadata analysis approaches for understanding and improving the functional involvement of rumen microbial consortium in digestion and metabolism of plant biomass. J. Genom..

[101] Matthews C., Crispie F., Lewis E., Reid M., O’Toole P.W., Cotter P.D. (2019). The rumen microbiome: A crucial consideration when optimising milk and meat production and nitrogen utilisation efficiency. Gut Microbes.

[102] Comtet-Marre S., Parisot N., Lepercq P., Chaucheyras-Durand F., Mosoni P., Peyretaillade E., Bayat A.R., Shingfield K.J., Peyret P., Forano E. (2017). Metatranscriptomics reveals the active bacterial and eukaryotic fibrolytic communities in the rumen of dairy cow fed a mixed diet. Front. Microbiol..

[103] Hook S.E., Wright A.-D.G., McBride B.W. Methanogens: Methane Producers of the Rumen and Mitigation Strategies. https://www.hindawi.com/journals/archaea/2010/945785/.

[104] Gilbert R.A., Townsend E.M., Crew K.S., Hitch T.C.A., Friedersdorff J.C.A., Creevey C.J., Pope P.B., Ouwerkerk D., Jameson E. (2020). Rumen virus populations: Technological advances enhancing current understanding. Front. Microbiol..

[105] Holman D.B., Gzyl K.E. (2019). A meta-analysis of the bovine gastrointestinal tract microbiota. FEMS Microbiol. Ecol..

[106] Callaway T.R., Dowd S.E., Edrington T.S., Anderson R.C., Krueger N., Bauer N., Kononoff P.J., Nisbet D.J. (2010). Evaluation of bacterial diversity in the rumen and feces of cattle fed different levels of dried distillers grains plus solubles using bacterial tag-encoded FLX amplicon pyrosequencing. J. Anim. Sci..

[107] Mao S., Zhang M., Liu J., Zhu W. (2015). Characterising the bacterial microbiota across the gastrointestinal tracts of dairy cattle: Membership and potential function. Sci. Rep..

[108] Bergmann G.T. (2017). Microbial community composition along the digestive tract in forage- and grain-fed bison. BMC Vet. Res..

[109] De Oliveira M.N.V., Jewell K.A., Freitas F.S., Benjamin L.A., Tótola M.R., Borges A.C., Moraes C.A., Suen G. (2013). Characterizing the microbiota across the gastrointestinal tract of a Brazilian Nelore steer. Vet. Microbiol..

[110] Malmuthuge N., Guan L.L. (2017). Understanding the gut microbiome of dairy calves: Opportunities to improve early-life gut health. J. Dairy Sci..

[111] Alipour M.J., Jalanka J., Pessa-Morikawa T., Kokkonen T., Satokari R., Hynönen U., Iivanainen A., Niku M. (2018). The composition of the perinatal intestinal microbiota in cattle. Sci. Rep..

[112] Morgavi D.P., Forano E., Martin C., Newbold C.J. (2010). Microbial ecosystem and methanogenesis in ruminants. Anim. Int. J. Anim. Biosci..

[113] Brashears M.M., Jaroni D., Trimble J. (2003). Isolation, selection, and characterization of lactic acid bacteria for a competitive exclusion product to reduce shedding of *Escherichia coli* O157:H7 in cattle. J. Food Prot..

[114] Wang L., Qu K., Li X., Cao Z., Wang X., Li Z., Song Y., Xu Y. (2017). Use of bacteriophages to control *Escherichia coli* O157:H7 in domestic ruminants, meat products, and fruits and vegetables. Foodborne Pathog. Dis..

[115] Henderson G., Cox F., Ganesh S., Jonker A., Young W., Janssen P.H. (2015). Rumen microbial community composition varies with diet and host, but a core microbiome is found across a wide geographical range. Sci. Rep..

[116] Wang O., McAllister T.A., Plastow G., Stanford K., Selinger B., Guan L.L. (2018). Interactions of the hindgut mucosa-associated microbiome with its host regulate shedding of *Escherichia coli* O157:H7 by cattle. Appl. Environ. Microbiol..

[117] Salaheen S., Kim S.W., Karns J.S., Hovingh E., Haley B.J., Van Kessel J.A.S. (2019). Metagenomic analysis of the fecal microbiomes from *Escherichia coli* O157:H7-shedding and non-shedding cows on a single dairy farm. Food Control.

[118] Zaheer R., Dugat-Bony E., Holman D., Cousteix E., Xu Y., Munns K., Selinger L.J., Barbieri R., Alexander T., McAllister T.A. (2017). Changes in bacterial community composition of *Escherichia coli* O157:H7 super-shedder cattle occur in the lower intestine. PLoS ONE.

[119] Xu Y., Dugat-Bony E., Zaheer R., Selinger L., Barbieri R., Munns K., McAllister T.A., Selinger L.B. (2014). *Escherichia coli* O157:H7 super-shedder and non-shedder feedlot steers harbour distinct fecal bacterial communities. PLoS ONE.

[120] Stenkamp-Strahm C., McConnel C., Magzamen S., Abdo Z., Reynolds S. (2018). Associations between *Escherichia coli* O157 shedding and the faecal microbiota of dairy cows. J. Appl. Microbiol..

[121] Sperandio V. (2010). SdiA sensing of acyl-homoserine lactones by enterohemorrhagic *E. coli* (EHEC) serotype O157:H7 in the bovine rumen. Gut Microbes.

[122] Sheng H., Nguyen Y.N., Hovde C.J., Sperandio V. (2013). SdiA aids enterohemorrhagic *Escherichia coli* carriage by cattle fed a forage or grain diet. Infect. Immun..

[123] Bertin Y., Chaucheyras-Durand F., Robbe-Masselot C., Durand A., De la Foye A., Harel J., Cohen P.S., Conway T., Forano E., Martin C. (2013). Carbohydrate utilization by enterohaemorrhagic *Escherichia coli* O157:H7 in bovine intestinal content. Environ. Microbiol..

[124] Bertin Y., Girardeau J.P., Chaucheyras-Durand F., Lyan B., Pujos-Guillot E., Harel J., Martin C. (2011). Enterohaemorrhagic *Escherichia coli* gains a competitive advantage by using ethanolamine as a nitrogen source in the bovine intestinal content. Environ. Microbiol..

[125] Bertin Y., Segura A., Jubelin G., Dunière L., Durand A., Forano E. (2018). Aspartate metabolism is involved in the maintenance of enterohaemorrhagic *Escherichia coli* O157:H7 in bovine intestinal content. Environ. Microbiol..

[126] Bertin Y., Deval C., De la Foye A., Masson L., Gannon V., Harel J., Martin C., Desvaux M., Forano E. (2014). The gluconeogenesis pathway is involved in maintenance of enterohaemorrhagic *Escherichia coli* O157:H7 in bovine intestinal content. PLoS ONE.

[127] Forano E., Chaucheyras-Durand F., Bertin Y., Martin C. (2013). EHEC carriage in ruminants and probiotic effects. Biol. Aujourdhui.

[128] Montagne L., Toullec R., Lallès J.P. (2000). Calf intestinal mucin: Isolation, partial characterization, and measurement in ileal digesta with an enzyme-linked immunosorbent assay. J. Dairy Sci..

[129] Snider T.A., Fabich A.J., Conway T., Clinkenbeard K.D.E. (2009). coli O157:H7 catabolism of intestinal mucin-derived carbohydrates and colonization. Vet. Microbiol..

[130] Aperce C.C., Heidenreich J.M., Drouillard J.S. (2014). Capacity of the bovine intestinal mucus and its components to support growth of *Escherichia coli* O157:H7. Anim. Int. J. Anim. Biosci..

[131] Segura A., Bertoni M., Auffret P., Klopp C., Bouchez O., Genthon C., Durand A., Bertin Y., Forano E. (2018). Transcriptomic analysis reveals specific metabolic pathways of enterohemorrhagic *Escherichia coli* O157:H7 in bovine digestive contents. BMC Genom..

[132] Zheng J., Wittouck S., Salvetti E., Franz C.M.A.P., Harris H.M.B., Mattarelli P., O’Toole P.W., Pot B., Vandamme P., Walter J. (2020). A taxonomic note on the genus *Lactobacillus*: Description of 23 novel genera, emended description of the genus *Lactobacillus* Beijerinck 1901, and union of *Lactobacillaceae* and *Leuconostocaceae*. Int. J. Syst. Evol. Microbiol..

[133] Morita M., Tanji Y., Mizoguchi K., Akitsu T., Kijima N., Unno H. (2002). Characterization of a virulent bacteriophage specific for *Escherichia coli* O157:H7 and analysis of its cellular receptor and two tail fiber genes. FEMS Microbiol. Lett..

[134] Shahrbabak S.S., Khodabandehlou Z., Shahverdi A.R., Skurnik M., Ackermann H.-W., Varjosalo M., Yazdi M.T., Sepehrizadeh Z. (2013). Isolation, characterization and complete genome sequence of PhaxI: A phage of *Escherichia coli* O157: H7. Microbiol. Read. Engl..

[135] Tomat D., Migliore L., Aquili V., Quiberoni A., Balagué C. (2013). Phage biocontrol of enteropathogenic and shiga toxin-producing *Escherichia coli* in meat products. Front. Cell. Infect. Microbiol..

[136] Lu Z., Breidt F. (2015). Escherichia coli O157:H7 bacteriophage Φ241 isolated from an industrial cucumber fermentation at high acidity and salinity. Front. Microbiol..

[137] Bumunang E.W., McAllister T.A., Stanford K., Anany H., Niu Y.D., Ateba C.N. (2019). Characterization of Non-O157 STEC infecting bacteriophages isolated from cattle faeces in North-West South Africa. Microorganisms.

[138] Montso P.K., Mlambo V., Ateba C.N. (2019). Characterization of lytic bacteriophages infecting multidrug-resistant Shiga toxigenic atypical *Escherichia coli* O177 strains isolated from cattle feces. Front. Public Health.

[139] Son H.M., Duc H.M., Masuda Y., Honjoh K.-I., Miyamoto T. (2018). Application of bacteriophages in simultaneously controlling *Escherichia coli* O157:H7 and extended-spectrum beta-lactamase producing *Escherichia coli*. Appl. Microbiol. Biotechnol..

[140] Stanford K., Bach S.J., Stephens T.P., McAllister T.A. (2010). Effect of rumen protozoa on *Escherichia coli* O157:H7 in the rumen and feces of specifically faunated sheep. J. Food Prot..

[141] Burow L.C., Gobius K.S., Vanselow B.A., Klieve A.V. (2005). A lack of predatory interaction between rumen ciliate protozoa and Shiga-toxin producing *Escherichia coli*. Lett. Appl. Microbiol..

[142] Paddock Z.D., Renter D.G., Shi X., Krehbiel C.R., DeBey B., Nagaraja T.G. (2013). Effects of feeding dried distillers grains with supplemental starch on fecal shedding of *Escherichia coli* O157:H7 in experimentally inoculated steers. J. Anim. Sci..

[143] Schneider L.G., Klopfenstein T.J., Stromberg Z.R., Lewis G.L., Erickson G.E., Moxley R.A., Smith D.R. (2018). A randomized controlled trial to evaluate the effects of dietary fibre from distillers grains on enterohemorrhagic *Escherichia coli* detection from the rectoanal mucosa and hides of feedlot steers. Zoonoses Public Health.

[144] Hallewell J., Barbieri L.R., Thomas J.E., Stanford K., McAllister T.A. (2013). Fecal shedding in cattle inoculated with *Escherichia coli* O157:H7 and fed corn or wheat distillers’ dried grain with solubles. J. Food Prot..

[145] Huws S.A., Kim E.J., Cameron S.J.S., Girdwood S.E., Davies L., Tweed J., Vallin H., Scollan N.D. (2015). Characterization of the rumen lipidome and microbiome of steers fed a diet supplemented with flax and echium oil. Microb. Biotechnol..

[146] Kasparovska J., Pecinkova M., Dadakova K., Krizova L., Hadrova S., Lexa M., Lochman J., Kasparovsky T. (2016). Effects of isoflavone-enriched feed on the rumen microbiota in dairy cows. PLoS ONE.

[147] McCabe M.S., Cormican P., Keogh K., O’Connor A., O’Hara E., Palladino R.A., Kenny D.A., Waters S.M. (2015). Illumina miseq phylogenetic amplicon sequencing shows a large reduction of an uncharacterised *Succinivibrionaceae* and an increase of the *Methanobrevibacter gottschalkii* clade in feed restricted cattle. PLoS ONE.

[148] Jiao S., Cao H., Dai Y., Wu J., Lv J., Du R., Han B. (2017). Effect of high-fat diet and growth stage on the diversity and composition of intestinal microbiota in healthy bovine livestock. J. Sci. Food Agric..

[149] Abdela N. (2016). Sub-acute Ruminal Acidosis (SARA) and its consequence in dairy cattle: A review of past and recent research at global prospective. Achiev. Life Sci..

[150] Petri R.M., Schwaiger T., Penner G.B., Beauchemin K.A., Forster R.J., McKinnon J.J., McAllister T.A. (2013). Characterization of the core rumen microbiome in cattle during transition from forage to concentrate as well as during and after an acidotic challenge. PLoS ONE.

[151] Petri R.M., Schwaiger T., Penner G.B., Beauchemin K.A., Forster R.J., McKinnon J.J., McAllister T.A. (2013). Changes in the rumen epimural bacterial diversity of beef cattle as affected by diet and induced ruminal acidosis. Appl. Environ. Microbiol..

[152] Fernando S.C., Purvis H.T., Najar F.Z., Sukharnikov L.O., Krehbiel C.R., Nagaraja T.G., Roe B.A., Desilva U. (2010). Rumen microbial population dynamics during adaptation to a high-grain diet. Appl. Environ. Microbiol..

[153] Neubauer V., Petri R., Humer E., Kröger I., Mann E., Reisinger N., Wagner M., Zebeli Q. (2018). High-grain diets supplemented with phytogenic compounds or autolyzed yeast modulate ruminal bacterial community and fermentation in dry cows. J. Dairy Sci..

[154] Tajima K., Aminov R.I., Nagamine T., Matsui H., Nakamura M., Benno Y. (2001). Diet-dependent shifts in the bacterial population of the rumen revealed with real-time PCR. Appl. Environ. Microbiol..

[155] Khafipour E., Li S., Plaizier J.C., Krause D.O. (2009). Rumen microbiome composition determined using two nutritional models of subacute ruminal acidosis. Appl. Environ. Microbiol..

[156] Plaizier J.C., Li S., Tun H.M., Khafipour E. (2017). Nutritional Models of Experimentally-Induced Subacute Ruminal Acidosis (SARA) differ in their impact on rumen and hindgut bacterial communities in dairy cows. Front. Microbiol..

[157] Khafipour E., Plaizier J.C., Aikman P.C., Krause D.O. (2011). Population structure of rumen *Escherichia coli* associated with subacute ruminal acidosis (SARA) in dairy cattle. J. Dairy Sci..

[158] Chaucheyras-Durand F., Faqir F., Ameilbonne A., Rozand C., Martin C. (2010). Fates of acid-resistant and non-acid-resistant Shiga toxin-producing *Escherichia coli* strains in ruminant digestive contents in the absence and presence of probiotics. Appl. Environ. Microbiol..

[159] McAllister T.A., Bach S.J., Stanford K., Callaway T.R. (2006). Shedding of *Escherichia coli* O157:H7 by cattle fed diets containing monensin or tylosin. J. Food Prot..

[160] Paddock Z.D., Walker C.E., Drouillard J.S., Nagaraja T.G. (2011). Dietary monensin level, supplemental urea, and ractopamine on fecal shedding of *Escherichia coli* O157:H7 in feedlot cattle. J. Anim. Sci..

[161] Cobbold R.N., Hancock D.D., Rice D.H., Berg J., Stilborn R., Hovde C.J., Besser T.E. (2007). Rectoanal junction colonization of feedlot cattle by *Escherichia coli* O157:H7 and its association with supershedders and excretion dynamics. Appl. Environ. Microbiol..

[162] Smith D., Blackford M., Younts S., Moxley R., Gray J., Hungerford L., Milton T., Klopfenstein T. (2001). Ecological relationships between the prevalence of cattle shedding *Escherichia coli* O157:H7 and characteristics of the cattle or conditions of the feedlot pen. J. Food Prot..

[163] Talley J.L., Wayadande A.C., Wasala L.P., Gerry A.C., Fletcher J., DeSilva U., Gilliland S.E. (2009). Association of *Escherichia coli* O157:H7 with filth flies (Muscidae and Calliphoridae) captured in leafy greens fields and experimental transmission of *E. coli* O157:H7 to spinach leaves by house flies (Diptera: Muscidae). J. Food Prot..

[164] Segura A. (2018). Portage Animal des Escherichia coli Entérohémorragiques: Colonisation et Interaction Avec le Microbiote Digestif Animal.

[165] Wray C., McLaren I., Pearson G.R. (1989). Occurrence of “attaching and effacing” lesions in the small intestine of calves experimentally infected with bovine isolates of verocytotoxic *E. coli*. Vet. Rec..

[166] Widiasih D.A., Matsuda I., Omoe K., Hu D.-L., Sugii S., Shinagawa K. (2004). Passive transfer of antibodies to Shiga toxin-producing *Escherichia coli* O26, O111 and O157 antigens in neonatal calves by feeding colostrum. J. Vet. Med. Sci..

[167] Rabinovitz B.C., Gerhardt E., Tironi Farinati C., Abdala A., Galarza R., Vilte D.A., Ibarra C., Cataldi A., Mercado E.C. (2012). Vaccination of pregnant cows with EspA, EspB, γ-intimin, and Shiga toxin 2 proteins from *Escherichia coli* O157:H7 induces high levels of specific colostral antibodies that are transferred to newborn calves. J. Dairy Sci..

[168] Rabinovitz B.C., Vilte D.A., Larzábal M., Abdala A., Galarza R., Zotta E., Ibarra C., Mercado E.C., Cataldi A. (2014). Physiopathological effects of Escherichia coli O157:H7 inoculation in weaned calves fed with colostrum containing antibodies to EspB and Intimin. Vaccine.

[169] Rugbjerg H., Nielsen E.M., Andersen J.S. (2003). Risk factors associated with faecal shedding of verocytotoxin-producing *Escherichia coli* O157 in eight known-infected Danish dairy herds. Prev. Vet. Med..

[170] Stenkamp-Strahm C., Lombard J.E., Magnuson R.J., Linke L.M., Magzamen S., Urie N.J., Shivley C.B., McConnel C.S. (2018). Preweaned heifer management on US dairy operations: Part IV. Factors associated with the presence of *Escherichia coli* O157 in preweaned dairy heifers. J. Dairy Sci..

[171] Johnson R.P., Cray W.C., Johnson S.T. (1996). Serum antibody responses of cattle following experimental infection with *Escherichia coli* O157:H7. Infect. Immun..

[172] Hoffman M.A., Menge C., Casey T.A., Laegreid W., Bosworth B.T., Dean-Nystrom E.A. (2006). Bovine immune response to shiga-toxigenic *Escherichia coli* O157:H7. Clin. Vaccine Immunol. CVI.

[173] Schaut R.G., Boggiatto P.M., Loving C.L., Sharma V.K. (2019). Cellular and mucosal immune responses following vaccination with inactivated mutant of *Escherichia coli* O157:H7. Sci. Rep..

[174] Schmidt N., Barth S.A., Frahm J., Meyer U., Dänicke S., Geue L., Menge C. (2018). Decreased STEC shedding by cattle following passive and active vaccination based on recombinant *Escherichia coli* Shiga toxoids. Vet. Res..

[175] Potter A.A., Klashinsky S., Li Y., Frey E., Townsend H., Rogan D., Erickson G., Hinkley S., Klopfenstein T., Moxley R.A. (2004). Decreased shedding of *Escherichia coli* O157:H7 by cattle following vaccination with type III secreted proteins. Vaccine.

[176] Van Donkersgoed J., Hancock D., Rogan D., Potter A.A. (2005). *Escherichia coli* O157:H7 vaccine field trial in 9 feedlots in Alberta and Saskatchewan. Can. Vet. J. Rev. Vet. Can..

[177] Torres A.G., Barrett A.D.T., Stanberry L.R. (2009). Chapter 51—Intestinal pathogenic *Escherichia coli*. Vaccines for Biodefense and Emerging and Neglected Diseases.

[178] Kropinski A.M., Lingohr E.J., Moyles D.M., Ojha S., Mazzocco A., She Y.-M., Bach S.J., Rozema E.A., Stanford K., McAllister T.A. (2012). Endemic bacteriophages: A cautionary tale for evaluation of bacteriophage therapy and other interventions for infection control in animals. Virol. J..

[179] Kieckens E., Rybarczyk J., De Zutter L., Duchateau L., Vanrompay D., Cox E. (2015). Clearance of *Escherichia coli* O157:H7 infection in calves by rectal administration of bovine lactoferrin. Appl. Environ. Microbiol..

[180] Ohya T., Marubashi T., Ito H. (2000). Significance of fecal volatile fatty acids in shedding of *Escherichia coli* O157 from calves: Experimental infection and preliminary use of a probiotic product. J. Vet. Med. Sci..

